# Downsizing the Channel Length of Vertical Organic Electrochemical Transistors

**DOI:** 10.1021/acsami.3c02049

**Published:** 2023-05-22

**Authors:** Jan Brodský, Imrich Gablech, Ludovico Migliaccio, Marek Havlíček, Mary J. Donahue, Eric D. Głowacki

**Affiliations:** † Bioelectronics Materials and Devices Lab, Central European Institute of Technology, Brno University of Technology, Purkyňova 123, 61200 Brno, Czech Republic; ‡ Institute of Scientific Instruments of the CAS, Královopolská 147, 61264 Brno, Czech Republic; § Department of Electrical and Electronic Technology, Faculty of Electrical Engineering and Communication, Brno University of Technology, 616 00 Brno, Czech Republic; ∥ Czech Metrology Institute, 638 00 Brno, Czech Republic; ⊥ Laboratory of Organic Electronics, ITN Campus Norrköping, Linköping University, SE-60174 Norrköping, Sweden

**Keywords:** vertical organic electrochemical transistor, microfabrication, PEDOT, electrochemical polymerization

## Abstract

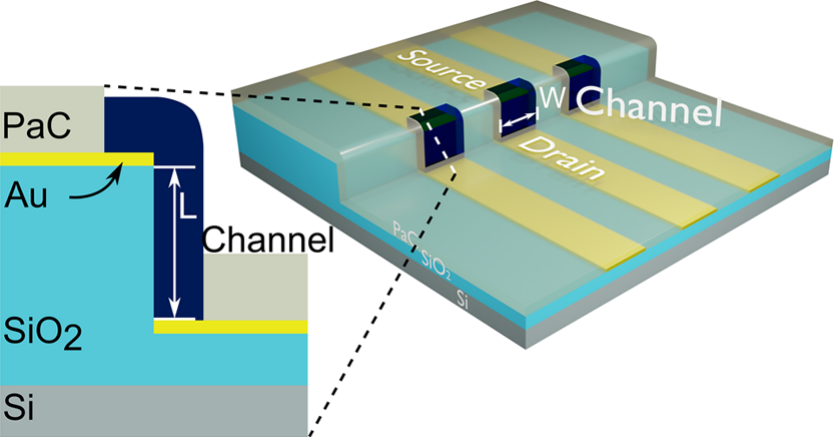

Organic electrochemical transistors (OECTs) are promising building blocks for bioelectronic devices such as sensors and neural interfaces. While the majority of OECTs use simple planar geometry, there is interest in exploring how these devices operate with much shorter channels on the submicron scale. Here, we show a practical route toward the minimization of the channel length of the transistor using traditional photolithography, enabling large-scale utilization. We describe the fabrication of such transistors using two types of conducting polymers. First, commercial solution-processed poly(dioxyethylenethiophene):poly(styrene sulfonate), PEDOT:PSS. Next, we also exploit the short channel length to support easy in situ electropolymerization of poly(dioxyethylenethiophene):tetrabutyl ammonium hexafluorophosphate, PEDOT:PF_6_. Both variants show different promising features, leading the way in terms of transconductance (*g*_m_), with the measured peak *g*_m_ up to 68 mS for relatively thin (280 nm) channel layers on devices with the channel length of 350 nm and with widths of 50, 100, and 200 μm. This result suggests that the use of electropolymerized semiconductors, which can be easily customized, is viable with vertical geometry, as uniform and thin layers can be created. Spin-coated PEDOT:PSS lags behind with the lower values of *g*_m_; however, it excels in terms of the speed of the device and also has a comparably lower off current (300 nA), leading to unusually high on/off ratio, with values up to 8.6 × 10^4^. Our approach to vertical gap devices is simple, scalable, and can be extended to other applications where small electrochemical channels are desired.

## 1 Introduction

Transistors are an essential component of modern electronics and have revolutionized the way in which we interact with technology. Although great advancements have been made in transistor technologies, further improvements in power consumption, device size, and overall performance are continuously sought. This is particularly true for relatively younger organic transistors when compared to their traditional inorganic counterparts. Organic transistors have gained attention as a promising technology for the development of low-cost, flexible, and large-area electronic devices.^1−3^ A subgroup of organic transistors, the organic electrochemical transistor (OECT), has emerged with particularly suitable characteristics for bioelectronic applications such as biosensors and biopotential recordings.^4−6^ OECTs are advantageous in bio-interfacing applications due to the mixed ionic/electronic conduction of their channel materials.^7−10^ This mixed conduction is ideal for ion-to-electron transduction, allowing for highly attainable amplification compared to inorganic or organic field effect transistors, providing quality biopotential recordings and good acquisition of small biosensor signals.^11−13^ Although applications such as these have benefited from OECT-related progress, further advances are needed to enable stable devices, high-density arrays, and complementary logic.^14−16^

When aiming to improve the amplification or speed properties of the OECT, the channel material and geometry are the main factors to consider.^17−19^ The OECT amplification, or transconductance (*g*_m_), is directly proportional to the electronic charge carrier mobility, μ, and the volumetric channel capacitance (C*)–intrinsic material properties. In contrast, its dependence on the channel width (*W*), thickness (*d*), and length (*L*) (i.e., the channel volume), *g*_m_ ∼ *Wd/L*, allows for manipulation through engineering approaches. Vertical organic electrochemical transistors (vOECTs) have been introduced as a straightforward method of reducing the physical device footprint and simultaneously significantly decreasing *L*, with the aim of enhancing the amplification properties.^20,21^ Although an increase in the overall channel volume generally improves *g*_m_, a trade-off exists when considering the transistor speed.^12^ This speed is particularly important for bioelectronic applications such as neural interfacing, where cutoff frequencies of up to at least 1 kHz are essential.^22^ The vOECT geometry facilitates reduced channel volumes while improving the *W*/*L* ratio, thus maintaining a good speed performance. Importantly, vOECTs also offer a useful geometry for electropolymerization, whereas planar devices typically result in poorly controlled, thick polymer film growth.^23−25^ Controlled electropolymerization of vOECT channels opens the door for the exploration of materials that are incompatible with solution-processing techniques and problematic for incorporation into typical OECT fabrication. Advanced geometries thus provide a means of improvement, not only in terms of transistor performance and reduced physical footprint but also in material investigation possibilities.

In this work, we demonstrate a straightforward fabrication technique for vOECTs, achieving highly reproducible channel geometries with an *L* value of 350 nm. The use of standard photolithography processes makes our approach widely applicable. High transconductance values of up to 52 and 68 mS are demonstrated for spin-coated PEDOT:PSS and electropolymerized PEDOT:PF_6_ channels, respectively. High on/off current ratios (≈8.6 × 10^4^) as well as useful cutoff frequencies (up to 2.1 kHz) are observed for bioelectronic applications. The vOECT approach developed in this work results in robust device structures, compatible with various channel material deposition methods, thus enabling the exploration of new materials.

## 2 Experimental Methods

### 2.1 Device Fabrication

**Figure 1:**
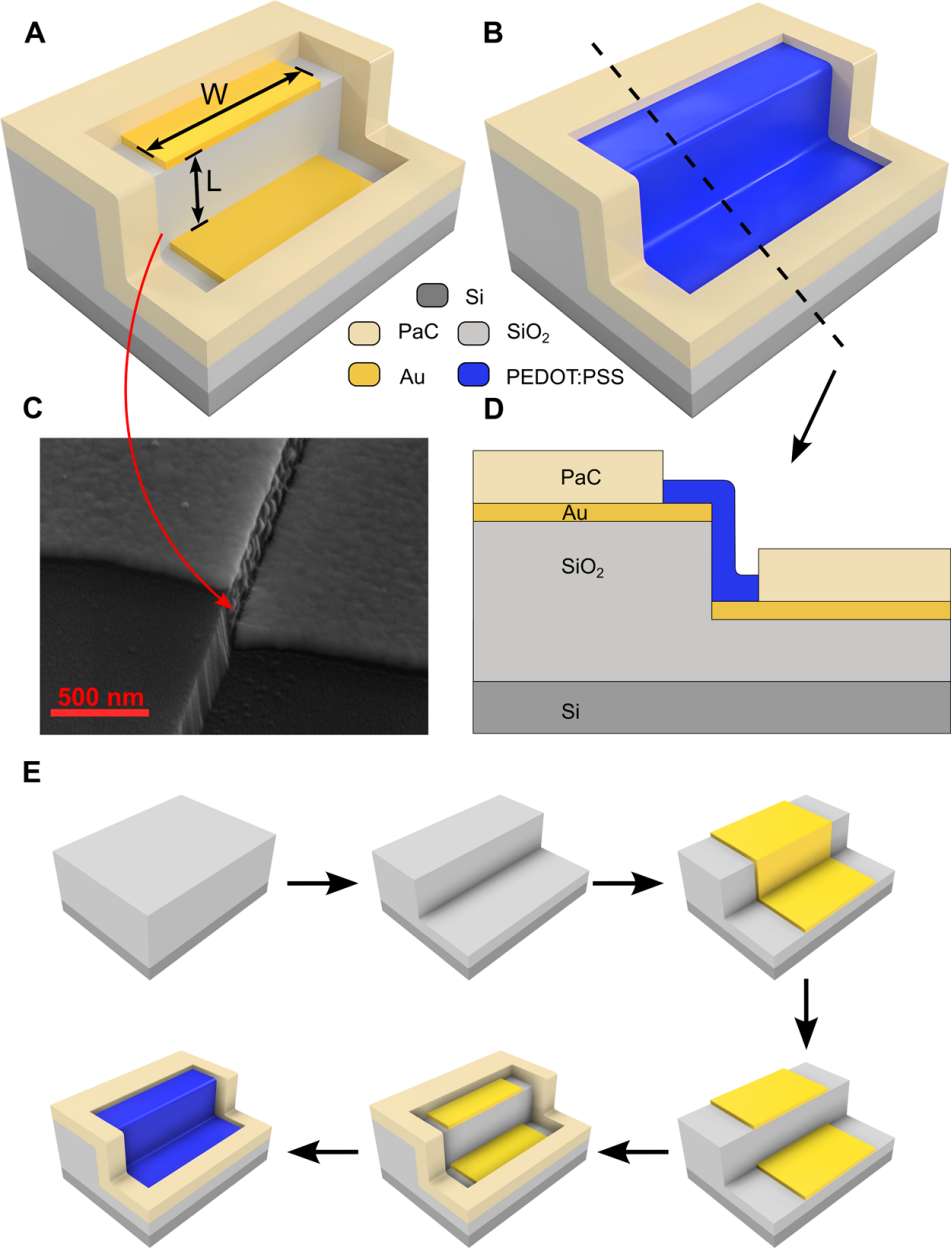
(A) Schematic of the device without the channel material, showing the geometry. (B) Device with spin-coated PEDOT:PSS. (C) SEM with a tilt of 50° of the fabricated channel area with the source and drain electrodes. (D) Cross section of the device. (E) Fabrication process schematic, starting with the Si/SiO_2_ substrate, followed by etching of the step in SiO_2_, patterning the electrodes and separating S/D, patterning the encapsulation, and creating the PEDOT:PSS channel.

Si wafers with a thermally grown SiO_2_ layer (525 ± 25 μm and 2.6 μm, respectively) were used as substrates. All AZ photoresists used in the fabrication were exposed through soda lime masks in a SÜSS MA8 mask aligner with an i-line filter, developed in AZ 726 MIF, and finally the photoresist was stripped in TechniStrip MLO-07 heated to 60 °C. In the first lithography step using AZ 701 MIR 29 cPs (4000 rpm, ≈1.5 μm, dose 225 mJ·cm^–2^), part of SiO_2_ was etched by capacitively coupled plasma reactive ion etching (CCP–RIE, CHF_3_/Ar 12/38 sccm, power = 200 W, pressure = 4 Pa, 580 V DC bias) to create the vertical step of the desired depth for the channel, partially defining the final *L* (Figure 1A). Ti/Au (3/100 nm) thin films were deposited with an electron beam evaporator (Bestec GmbH) and patterned with wet etching using an AZ 1514H photoresist mask (4000 rpm, ≈1.4 μm, dose 110 mJ·cm^–2^) and KI/I_2_ and HF:HNO_3_:H_2_0 (1:1:100) etchants for Au and Ti etching, respectively. Even though the sidewall between the electrodes is almost perpendicular, the electrodes were partially shorted due to the ultra-thin deposit of Ti/Au on the sidewall. At this point, the source and drain electrodes were fully separated at the previously created step using an ion-milling instrument (Scia Systems GmbH) equipped with a three-grid ion beam optics and a space charge neutralizer. A collimated Ar^+^ ion beam with an energy of 600 eV (ion beam current = 200 mA) was used to impact the substrate, at a small angle of 25° with respect to the substrate, to sputter the metals at the vertical sidewall, while the wafer surface etching rate is slower,^26^ resulting in the device shown in Figure 1C.

**Figure 2:**
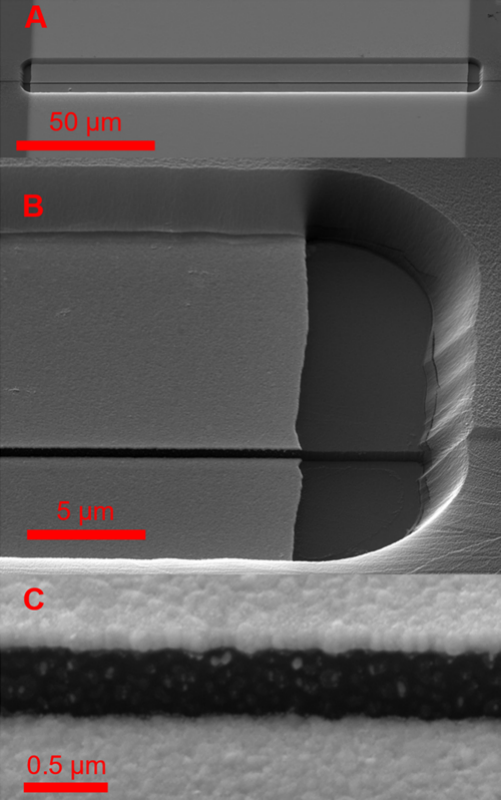
Tilted SEM images of the device with increasing magnification: (A) whole vOECT structure with a tilt of 50°; (B) detail of vOECT structure showing the step between the source and drain electrodes and the encapsulation layer; (C) high-magnification view of the step in between the source and drain electrodes.

Afterward, we used an SCS Labcoater with a Silane A-174 adhesion promoter in the chamber to deposit the 3 μm thick parylene-C encapsulation layer. In the next step, we opened the contact pads and channels using a thick AZ 1518 photoresist and O_2_ plasma (200 W, 13.3 Pa, O_2_ 50 sccm, 450 V DC bias) in the CCP–RIE system. One substrate was diced using the dicing saw to single (15 × 15) mm^2^ chips, later used for the electrochemical polymerization of PEDOT:PF_6_. A device without the polymer channel is shown in Figure 2. On the substrate intended for the spin-coating of the channel material, before depositing a sacrificial parylene-C layer with a thickness of 2 μm, a dilute solution of anti-adhesive soap was spin-coated at 1000 rpm (2% V/V Micro90). With the use of an AZ 12XT-20PL-10 photoresist and RIE, the channel area was opened and prepared for spin-coating of a PEDOT:PSS solution.

### 2.2 Spin-Coating and Electrodeposition of PEDOT

To create the transistor channel with PEDOT:PSS, a dispersion of PEDOT:PSS (Clevios PH 1000, Heraeus Holding GmbH) with 5 wt % ethylene glycol, 0.1 wt % dodecyl benzene sulfonic acid, and 1 wt % of (3-glycidyloxypropyl)-trimethoxysilane (GOPS) was spin-coated on the substrate at 650 rpm to a thickness of 400 nm, determined from a test peel-off using a profilometer and AFM measurements. The substrate was then prebaked at 90 °C for 2 min, and the PEDOT:PSS layer was patterned by peeling off the parylene-C sacrificial layer. A subsequent annealing step at 140 °C for 45 min was performed to cross-link the layer. The substrate was then placed in deionized (DI) water overnight to remove the low-molecular-weight compounds embedded in the organic layer.

To electrochemically polymerize PEDOT:PF_6_ on the vOECT channel, 3,4-ethylenedioxythiophene (EDOT), tetrabutylammonium hexafluorophosphate (TBAPF_6_), and acetonitrile (CH_3_CN) were purchased from Sigma-Aldrich. In order to better control the thickness of the layer, EDOT solutions were prepared with two different concentrations of 1 mM and 5 mM with 100 mM of TBAPF_6_ in CH_3_CN. Electrochemical polymerization was carried out using a galvanostatic method, varying both the applied current and time, to attain different polymer thicknesses, as shown in Figure 4B. With an Ivium PocketSTAT2 potentiostat, a two-electrode configuration was used, where the source and drain electrodes were shorted to function as a working electrode, and a commercial Pt wire embedded in a modified syringe containing electrolyte was utilized as the counter electrode. A small drop of the electrolyte was placed on the open channels, while the source and drain electrodes were contacted with micromanipulator probes.

### 2.3 Characterization

An optical microscope (Zeiss Axio Imager A2) and a scanning electron microscope (Tescan Mira3) were used to observe the devices during and after the fabrication process. To electrically characterize the devices, a probe station with a stereomicroscope was employed. A PDMS well was used to hold a volume of 100 mM KCl, in which an Ag/AgCl gate electrode was immersed. To capture the steady-state characteristics and temporal response, a Keithley 4200A-SCS parameter analyzer was used. The frequency response was obtained by connecting the vOECT as a simple voltage amplifier, with a series drain load resistor *R*_L_ = 1 kΩ. The drain–source voltage (*V*_DS_) was set by a Keysight U2722A source measurement unit, and the output signal was captured by a digital oscilloscope Keysight DSOX2004A with a built-in sine-wave generator, which was used for the gate–source voltage *V*_GS_ control. We applied a sine wave of *V*_GS_ = 20 mV peak-to-peak, with a DC voltage offset, to work in the regime of maximum *g*_m_. This offset was set individually for each channel and was typically in range from 0 to 200 mV.

The polymer channel thickness was determined by the use of a stylus profilometer DektakXT (Bruker) and verified by an atomic force microscope (Dimensions Icon, Bruker). DektakXT was set to make a 100 μm long scan, while the applied force on the tip with a radius of 2.5 μm was set to the lowest possible value of ≈9.8 mN. At this point, we observed scratches only in the electropolymerized layers after the stylus profilometer measurement; therefore, we decided to verify and solve this issue using AFM, which is gentler to the materials with low hardness. We chose the tapping mode with the RTESPA-525 probe for this purpose.

## 3 Results and Discussion

**Figure 3:**
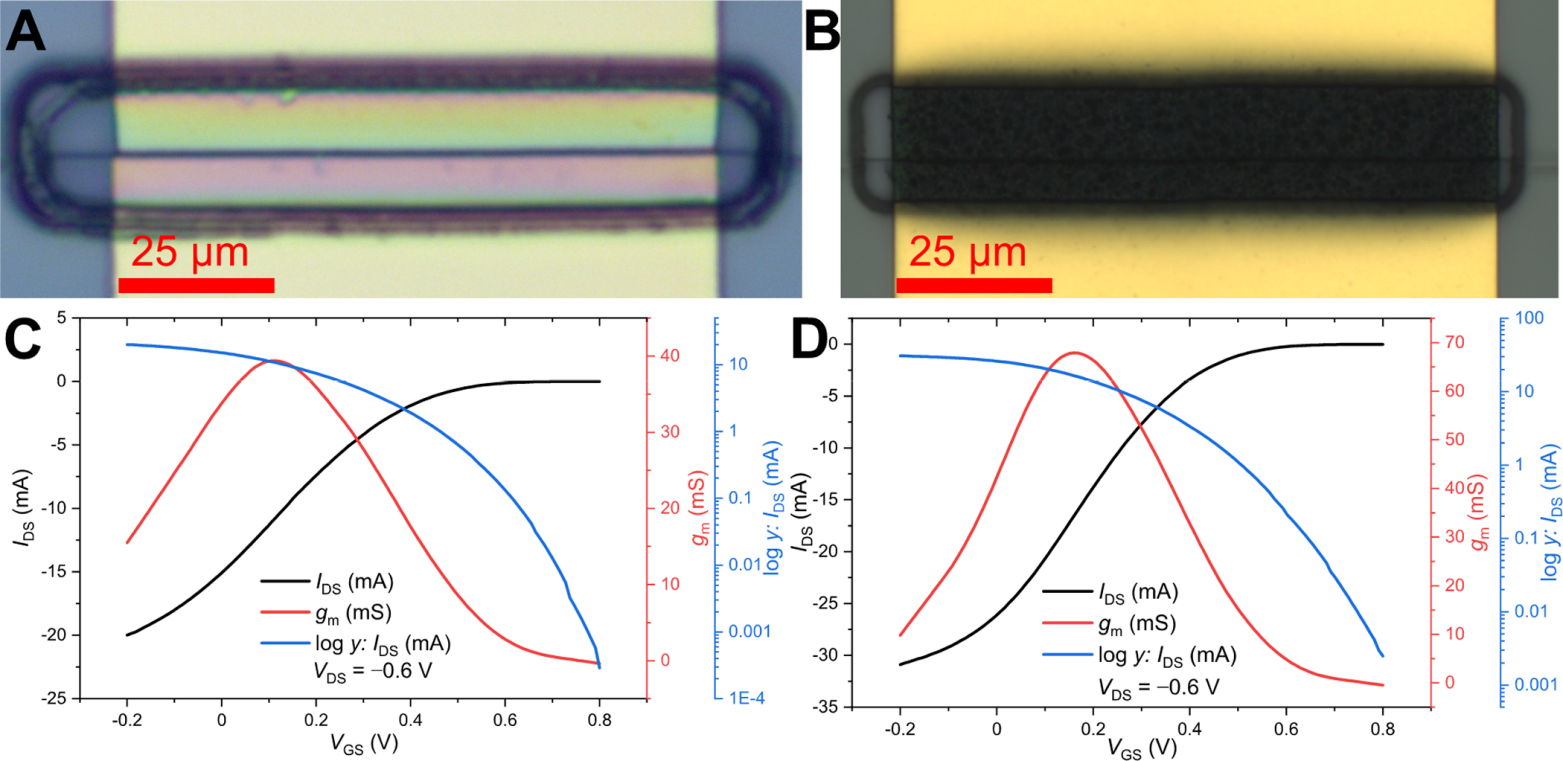
Optical micrographs and the corresponding transfer characteristics of (A,C) spin-coated PEDOT:PSS device with the channel width *W* = 100 μm and thickness *d* = 400 nm. (B,D) Electropolymerized PEDOT:PF_6_, with *W* = 100 μm and *d* = 280 nm.

All fabricated devices had the same *L* of 350 nm. The transistor channel *W* was varied with values of (50, 100, and 200) μm, with the first two used most often in this work. As mentioned in Experimental Methods, two distinct sample types were fabricated to make a side-by-side comparison of spin-coated and electropolymerized channels. Two devices of similar thickness are shown in Figure 3A,B, with their corresponding transfer characteristics and transconductances. The spin-coated PEDOT is clearly more transparent and uniform. It can be noted that the peak *g*_m_ is higher for the electropolymerized (68 mS) than the spin-coated PEDOT (38 mS). This result, however, comes at the cost of higher (≈2 μA) off current (*I*_OFF_) of the electropolymerized device, as is visible in the logarithmic (blue line) scale of *I*_DS_ in Figure 3C,D. On the contrary, the spin-coated PEDOT:PSS benefits from the short channel *L*, showing high *I*_ON_ of ≈20 mA and a low *I*_OFF_ of only ≈290 nA. The peak *g*_m_ also shifts noticeably more toward positive *V*_GS_ for the electropolymerized device.

**Figure 4:**
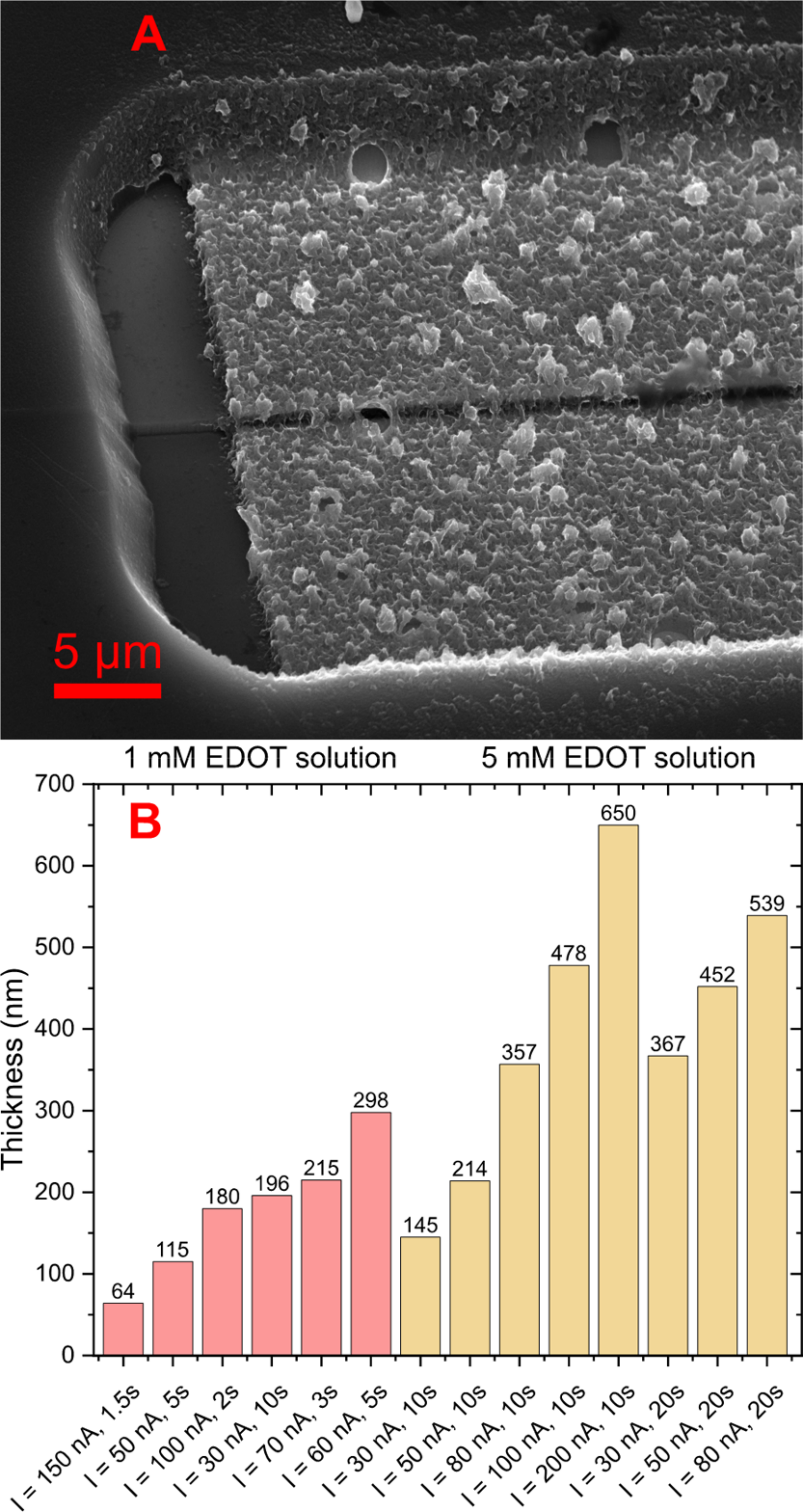
(A) SEM image of the device with ≈360 nm thick electropolymerized PEDOT:PF_6_ channel. (B) Thickness of the electropolymerized channel based on the process parameters.

Another important feature which plays a fundamental role during the polymer electrodeposition is the charge consumed for the growth of the channel, defined as the current applied galvanostatically over a fixed range of time. It is important to optimize and reproduce these values in order to use them as a trustworthy and reliable source of information for the following depositions. The concentration of monomer in the solution used for the electropolymerization also dictates the final thickness of the polymer. This is the major reason for which the concentration of the monomer was reduced five times (from 5 to 1 mM). This provides reasonable thicknesses, useful for comparisons and with accessible values of fixed charges during the deposition procedure. Initially, the experiments were carried out using 5 mM solutions of EDOT, resulting in less control over the film growth for thinner (<150 nm) layers (unreliable results were observed for each experiment despite maintaining the same setup and using fresh solutions for each deposition). Both for the high and low amounts of charge during deposition, the deposited layer thickness was very inconsistent. This issue was not observed for thicker (>150 nm) layers. On the other hand, when using a fresh solution of 1 mM monomer concentration and optimal parameters, it was possible to coat the surface of the electrodes quite uniformly and obtain thin layers, as shown in the SEM image in Figure 4A. To show the repeatability of the layer thickness with the 1 mM solution, a set of parameters was used twice, and the final thickness was checked by a profilometer.

**Figure 5:**
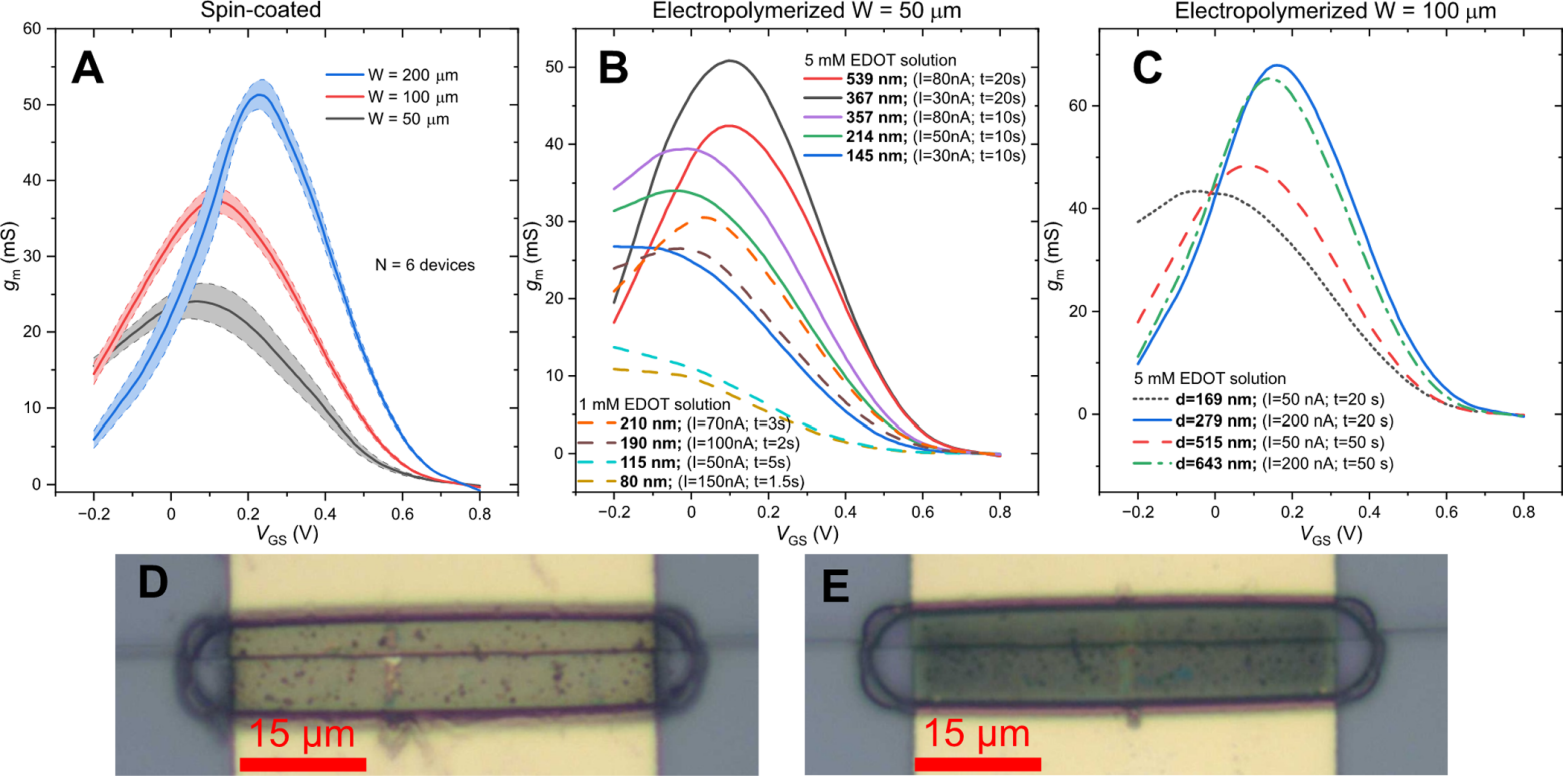
Transconductance (*g*_m_) of (A) spin-coated PEDOT:PSS with thickness *d* = 400 nm and varied channel width *W*. (B,C) Electropolymerized PEDOT:PF_6_ with varied *d* and *W* of 50 and 100 μm, respectively. (D,E) Optical microscopy image of the electropolymerized channel with *d* of 80 and 200 nm.

Transconductance curves of all fabricated devices were acquired. For spin-coated PEDOT:PSS, the number of measured devices (*N*) for each channel width was 6, with the mean value and standard deviation shown in Figure 5A. An increase in performance is apparent with wider channels, while the deviation between individual devices is quite small. The transconductance curves of electropolymerized layers are displayed in Figure 5B,C. A trend of increasing performance with the increasing thickness of PEDOT:PF_6_ is mostly seen, as it was expected; however it may be noted that there are few outliers. This suggests that not only the thickness but the way the channel was grown also influences the performance of the device. Figure 5D,E shows the electropolymerized layers with *d* of 80 and 200 nm. It can be seen that at lower thicknesses, the layer is still somewhat transparent, though not as much as spin-coated PEDOT:PSS. Crystal formation from the electropolymerization solution is also visible, despite the fact that active measures were taken to avoid the evaporation of the solution during the deposition process and that the devices were washed thoroughly in clean acetonitrile afterward.

**Figure 6:**
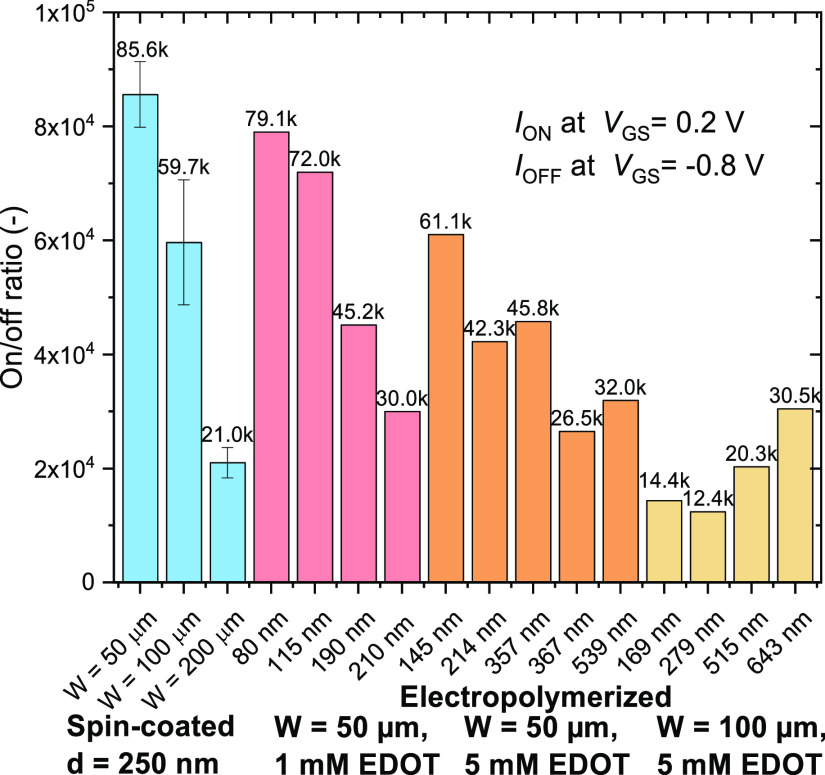
On/off ratio of the fabricated devices, with *y*-error for the spin-coated devices (*N* = 6).

The transistor performance demonstrates the benefits from the short channel geometry, also in terms of the on/off ratio, yielding relatively high values not usually achieved with PEDOT. As shown in Figure 6, spin-coated PEDOT:PSS performs the best with a value of ≈8.6 × 10^4^. The ratio diminishes as *W* increases, although it remains of the same magnitude. With the electropolymerized devices, the ratio clearly scales down with the thickness of the layer; however, for *W* = 100 μm, this trend is broken, suggesting again that the performance of those layers is dependent on the way they were grown.

**Figure 7:**
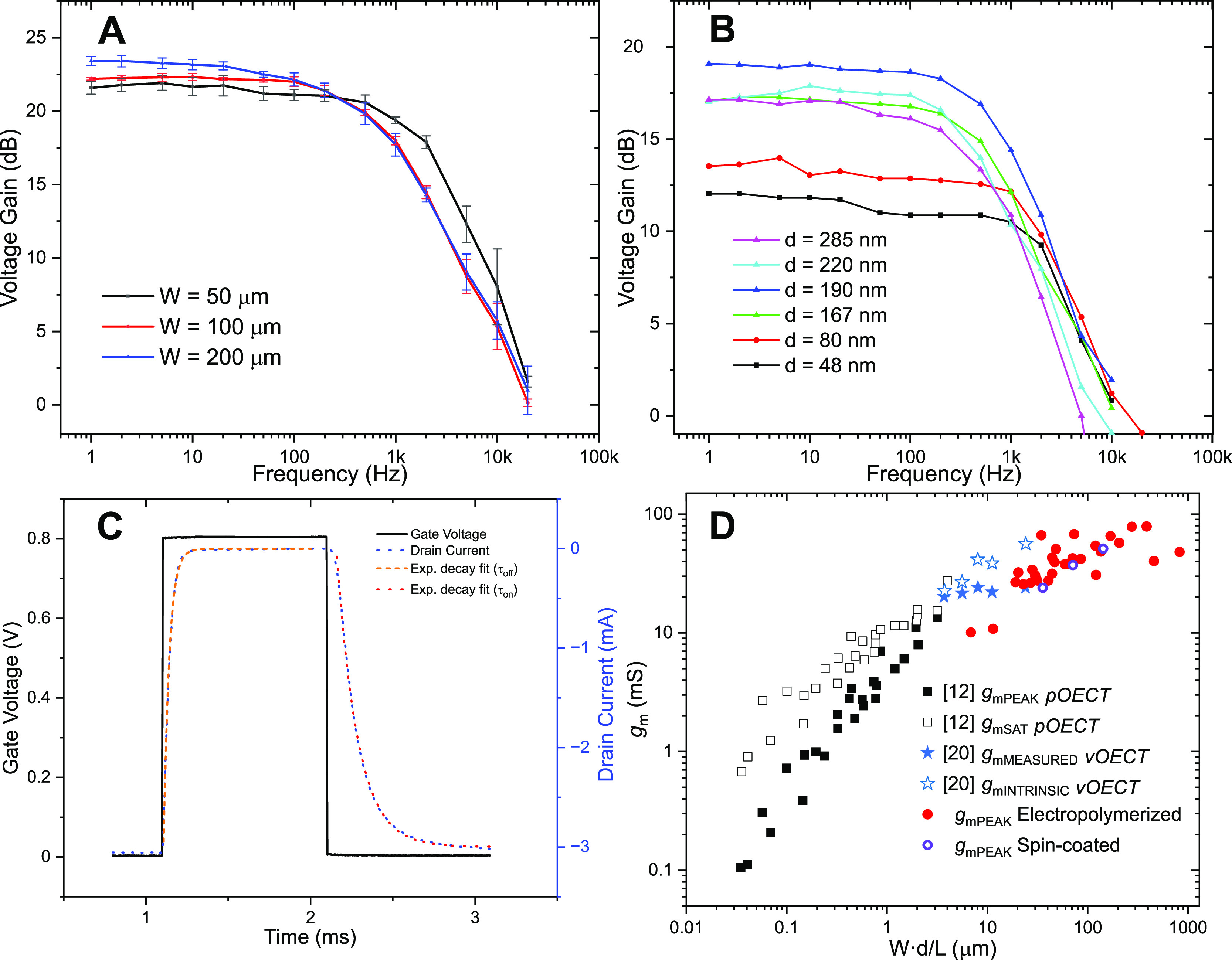
Frequency response of (A) spin-coated PEDOT:PSS with a thickness (*d*) of 400 nm. (B) Electropolymerized PEDOT:PF_6_ with a constant channel width (*W*) of 50 μm, with *d* as a varied parameter. (C) Temporal response of the spin-coated device (*W* = 50 μm, *d* = 400 nm, and *V*_DS_ = −0.6 V), with extracted time constants of τ_OFF_ ≈ 36 μs and τ_ON_ ≈ 124 μs. (D) Transconductance figure-of-merit plot comparing the data shown in a previous work with planar^12^ and vertical^20^ OECTs (pOECT, vOECT) to the data obtained in this work (red and violet circles).

From the frequency response at maximum *g*_m_ (Figure 7A,B), the cutoff frequency (*f*_T_) of all devices was extracted and is shown in Table 1. It clearly scales down with increasing *W* or *d* of the transistor. Due to the short channel *L* and minimized overlap of PEDOT with Au electrodes, which is in total ≈6 μm, competitive values (for the given channel volumes) of *f*_T_ were obtained.

**Table 1:** Values of Cutoff Frequencies of Fabricated Devices

***W* (μm)**	50	100	200	50					
***d* (nm)**	400			48	80	167	190	220	285
***f*_T_ (Hz)**	1230	610	330	1824	2110	510	495	420	375

The spin-coated device performs better than the electropolymerized ones in terms of the frequency response; therefore, a temporal response of six identical devices was captured as well, yielding averaged time constants of τ_OFF_ = (36.4 ± 1.8) μs and τ_ON_ = (124.0 ± 1.9) μs for devices with *W* = 50 μm and *d* = 400 nm (Figure 7C). The time constant for turning ON the transistor is significantly slower, showing the same behavior as reported in some other works.^27−30^ Paudel et al.^27^ showed that for a planar OECT, lateral current in the channel when switching the transistor off is the limiting factor, as the channel length is usually much larger than the thickness of the semiconductor, rendering turning OFF the slower process of the two. Here, we attribute the opposite behavior partly to the fact that the channel length and semiconductor thickness are similar. The second factor could be an increase in the ionic resistance, slowing the switching to the ON state. We extracted the volumetric capacitance (C*) from EIS according to a recent review^15^ and obtained estimated values of 154 and 282 F·cm^–3^ for PEDOT:PSS and PEDOT:PF_6_, respectively. We have to note that the volume determination in our electropolymerized channel is not as straightforward as in the case of planar OECT due to significant disproportions between the channel area and overlap on the electrode, as well as rougher morphology; however, it is in any case clear that the electropolymerized PEDOT capacitance is higher, explaining the difference in speed. To make a comparison with a previous work, a transconductance figure-of-merit plot is shown in Figure 7D. Transconductance of planar OECTs^12^ (black and white squares) increases linearly with the *Wd/L* ratio. As the downscaling of *L* leads to higher values of the ratio, a deviation from the linear trend can be observed, continuing the transconductance growth with a decreased slope of the fit.

## 4 Conclusions

In this work, we described a vOECT fabrication method that is scalable, reliable, and uses conventional microfabrication techniques, obtaining a submicrometer channel length without the need for electron beam lithography. The channel length can be precisely tuned to desired values in lower hundreds of nanometers. As the source and drain electrodes do not overlap, the parasitic properties are reduced, and it is possible to further downscale the channel length to tens of nanometers. Compared to other works, the actual organic layer creating the transistor channel is prepared as the last step, limiting its exposure to undesired contamination or damage from the fabrication process. Finally, the fact that the method is intrinsically compatible with silicon wafer processing means straightforward integration with a silicon circuit. This can be used to make powerful integrated sensors or amplifiers, combining the strengths of mixed ionic–electronic conductor ECTs with silicon CMOS computing. Two different approaches were taken to create the transistor channel, first was the spin-coated PEDOT:PSS, while the second type was electropolymerized PEDOT:PF_6_. High peak transconductance was obtained for both types. The spin-coated devices had a maximum transconductance of 52 mS, with the preserved speed parameters of the transistor (τ_OFF_ ≈ 36 μs and τ_ON_ ≈ 124 μs) and an exceptional on/off current ratio of ≈8.6 × 10^4^. The electropolymerized devices have shown a higher maximum transconductance of 68 mS for layers of similar thickness. However, their speed of operation was considerably slower, with approximately 3 × lower *f*_T_. This could be due to the higher density of the electropolymerized PEDOT and/or due to the lack of PSS-rich phase present in the spin-coated version. Essentially, we have shown a simple platform that could be used for the research of newly synthesized channel materials, regardless of their patterning method. The electropolymerization process, especially, can be quite easily modified, as its parameter space is very vast. The control of morphology could be advantageous for specific applications. Our platform can also be easily translated to flexible organic bioelectronic substrates (i.e., parylene-C and polyimide) designing predefined trenches mimicking the same step made here in SiO_2_. An important conclusion is that our findings add to the growing body of recent research^15,21^ which show that achieving cutoff frequencies in the range above 1 kHz is challenging and that limitations are likely intrinsic to the conducting polymer itself, not the geometric structure.
